# Metamaterial study of quasi-three-dimensional bowtie nanoantennas at visible wavelengths

**DOI:** 10.1038/srep41966

**Published:** 2017-02-08

**Authors:** Yukun Zhao, Feng Yun, Yi Huang, Shuai Wang, Lungang Feng, Yufeng Li, Maofeng Guo, Wen Ding, Ye Zhang

**Affiliations:** 1 Key Laboratory of Physical Electronics and Devices of Ministry of Education and Shaanxi Provincial Key Laboratory of Photonics & Information Technology, Xi’an Jiaotong University, Xi’an, Shaanxi 710049, P. R. China; 2 Solid-State Lighting Engineering Research Center, Xi’an Jiaotong University, Xi’an, Shaanxi 710049, P. R. China; 3 Department of Electrical Engineering and Electronics, University of Liverpool, Liverpool L69 3GJ, United Kingdom

## Abstract

In this paper, a novel array of quasi-three-dimensional (quasi-3D) bowtie nanoantennas has been investigated numerically and experimentally. A low-cost and facile method has been designed and implemented to fabricate the quasi-3D bowtie nanoantennas. The fabrication processes containing laser patterning and wet etching have demonstrated the advantages of easily tuning the periodic and diameter of microhole arrays. According to the simulated results, the electric and magnetic resonances at visible wavelengths are obtained in the tips and contours of the metamaterials made of the quasi-3D bowtie nanoantennas, respectively. The effects of the size and gap of quasi-3D bowtie nanoantennas on the array performance have also been studied. The underlying mechanism suggests that different electric and magnetic resonant ranges of the metamaterials could contribute to the broad resonant range for the monolithic metamaterials.

Electromagnetic (EM) metamaterials are artificially designed media, exhibiting unique EM properties that are unattainable with natural materials^1^,^2^,^3^. Due to many applications in the fields of negative refractive index, invisibility cloaking, perfect lensing and superconductors, metamaterials have become one of the hottest fields of photonics^4^,^5^,^6^,^7^. According to the effective medium theory of metamaterials, the interior structure characteristic size of the medium must be in the nanometer scale when operating at visible wavelengths^8^,^9^. As experimentally fabricating of nanoscale metamaterials is one of the severe challenges, especially for fabrications with low-cost and facile processes, metamaterials have been limited to visible wavelengths^2^,^10^,^11^. Thus, very few phenomena theoretically proposed for metamaterials were confirmed at visible wavelengths^2^,^12^,^13^.

For practical applications of metamaterials, three-dimensional or even bulk structures are needed^5^,^14^,^15^. Scaling down three-dimensional (3D) metamaterials for applications at higher frequencies in the infrared and visible regimes remains an active focus of research^11^,^16^. The key motivation in producing 3D structures has always been the realization of metamaterials with effective constituent properties that can be tuned in all propagation directions over a wide range^16^. By numerical simulations, M. Giloan *et al*. have reported that 2D bowtie nanoantennas can be utilized as metamaterials in the visible range^8^. Magnetic fields of 2D bowtie nanoantennas have also been studied by some groups^17^,^18^,^19^. Although plasmonic nanoantennas can create highly enhanced local fields when pumped resonantly and modify the optical wavefront within an extremely thin layer^20^,^21^,^22^, it is still challenging to realize magnetic response at visible wavelengths, especially in the 3D space^23^. In other words, 3D or quasi-3D bowtie nanoantennas could be a promising route to realize 3D metamaterials at visible wavelengths. They could also be utilized in many other fields, including the optical antennae, optical devices, sensing, energy harvesting and nanotweezers^17^,^19^,^22^,^24^.

3D metamaterials can be achieved using a “top-down” or a “bottom-up” fabrication approach. However, “top-down” fabrication approaches such as electron-beam lithography and focused ion-beam technology require expensive apparatuses and complicated processes^8^,^23^,^25^, which significantly limited the promotion of 3D metamaterials. Therefore, an effective fabrication approach with low-cost and facile processes is still challenging but very attractive and promising for nanostructures applied in visible regions, especially for 3D metamaterials. However, so far, very few papers have been published in the field of simplifying the fabrication processes of 3D or quasi-3D bowtie nanoantennas and utilizing these nanostructures as metamaterials.

In recent years, laser direct writing process has become a popular tool to fabricate arbitrary microstructures^15^,^26^,^27^. By the laser patterning, our previous works have fabricated microhole arrays on the ceramic phosphor platelets and sapphires^28^,^29^. Combined with wet etching, the laser patterning can also be attractive because of its relatively simple devices, high fill factor of the lenses, and flexibility for regulating the curvature^27^. Furthermore, 2D bowtie nanoantennas have been investigated in our previous work^30^. However, up to now, combining laser patterning and wet etching to fabricate 3D or quasi-3D bowtie nanoantennas is yet to be implemented. In this paper, a facile and low-cost method with laser patterning and wet etching has been designed to fabricate quasi-3D bowtie nanoantennas. EM properties of metamaterials made of the quasi-3D bowtie nanoantennas have been investigated.

## Results

### Design and numerical simulations

**Figure 1:**
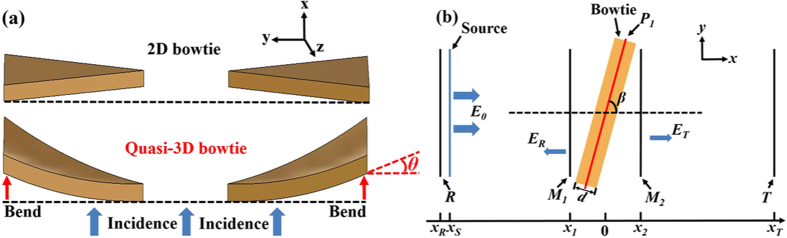
(**a**) Schematic of quasi-3D bowtie nanoantennas and (**b**) numerical setup in FDTD models used for the metamaterial slab made of bowtie nanoantennas.

Conventional 2D bowtie nanoantennas are often fabricated in a single flat plane^8^,^21^,^22^, like the y-z plane in Fig. 1(a). When 2D bowtie nanoantennas are bent or varied along the third coordinate axis [x axis in Fig. 1(a)], quasi-3D bowtie nanoantennas could be achieved. As illustrated in Fig. 1(a), the symbol “*θ*” represents the bending angle between the tangent line of quasi-3D bowtie nanoantennas and the horizontal line. The bent bowtie nanoantennas (quasi-3D bowtie nanoantennas) may be capable of bending light in three directions^20^,^24^, which is significant for fabricating 3D metamaterials^16^. Figure 1(b) illustrates the schematic of the configuration often used in a numerical setup. 3D finite-difference time-domain (FDTD) models using commercial software *FDTD Solutions* are utilized to investigate the EM properties of metamaterials made of quasi-3D bowtie nanoantennas. In the simulation models, the refractive index of the background (*n*) and slab thickness (*d*) are set to be 1.0 and 30 nm, respectively. The plane waves having the wavelength range of 500–1500 nm are used as the light source, which are placed at the position *X*_*S*_ in front of the bowtie slab [Fig. 1(b)]. In the simulation models, the symbol “*β*” represents the angle between the polarization of plan waves and metamaterial slab fabricated of bowtie nanoantennas. For the incident EM waves propagating from a direction, such as from the bottom, different parts of quasi-3D bowtie nanoantennas may have different angles of *β*, which may result in different resonant couplings in the 3D space.

The reflection and transmission parameters are calculated at the front (*M*_*1*_) and back (*M*_*2*_) faces of the metamaterial slab, respectively^8^. The red line (*P*_*1*_) shows the position for monitoring the electromagnetic field of slab [Fig. 1(b)]. The complex values of reflected (*E*_*R*_) and transmitted (*E*_*T*_) electric fields are evaluated in *R* and *T* planes situated at the positions *X*_*R*_ and *X*_*T*_ in front and behind the slab, respectively^8^. They are set as *X*_*R*_ = −480 nm and *X*_*T*_ = 480 nm in the models, considering the magnitude and phase distribution in R and T planes. Some other positions are set as *X*_*S*_ = −480 nm, *X*_*1*_ = −192 nm and *X*_*2*_ = 192 nm, where *X*_*S*_, *X*_*1*_ and *X*_*2*_ are the x-coordinates of planes source, *M*_*1*_ and *M*_*2*_ in Fig. 1(b), respectively. The transmission parameters at M_2_ plane (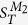) are related to the incident, reflected and transmitted electromagnetic fields^8^,^31^. The transmission spectra at M_2_ plane (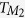) are calculated as:


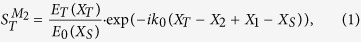



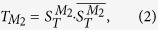


### Optical microscope images of the fabricated samples

**Figure 2:**
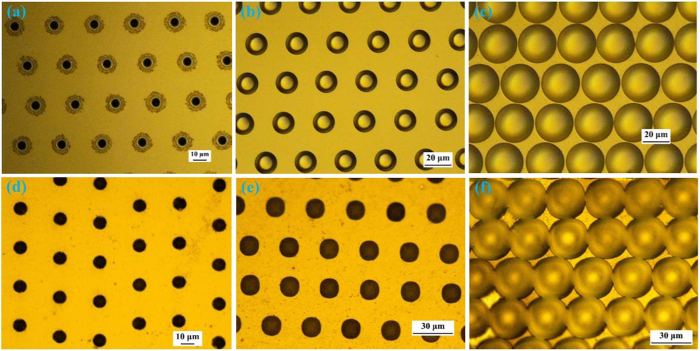
Experimental top-view OM images of microholes etched approximately (**a**) 0 min, (**b**) 5 min, and (**c**) 13 min on the glass substrate. Experimental top-view OM images of microholes etched approximately (**d**) 0 min, (**e**) 3 min, and (**f**) 18 min on the Si substrate.

Figure 2 is a collection of the optical microscopy (OM) surface micrographs, which indicates that hexagonally-ordered microholes can be obtained on both glass and Si substrates. It can be clearly seen from Fig. 2(a,b,d) and (e) that, at the beginning of the etching process, a short time is required for the acid to expand and smooth the laser-irradiation-induced microholes to form concave structures on both glass and Si substrates. Figure 2(c) indicates that uniform microhole distributions can be achieved on the glass substrates, and Fig. 2(f) shows those on the Si substrates. With the etching time increasing, the diameters of microholes enlarge and inner surfaces of microholes become smoother.

**Figure 3:**
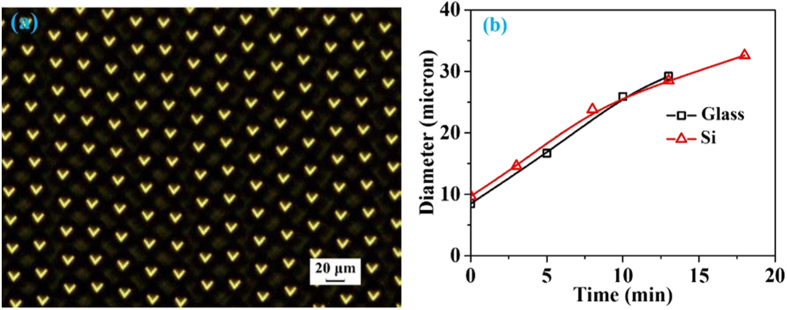
(**a**) Experimental top-view OM images of microholes etched approximately 13 min on the glass substrate. The image of the symbol “∨” generated through the fabricated microhole array. (**b**) The evolution of the diameter versus the wet-etching time on different substrates.

Figure 3 illustrates the experimental top-view OM images of microholes and the evolution of diameters under different wet-etching times. A sheet of paper with a word “∨” is placed between the light source and fabricated microhole arrays, and Fig. 3(a) is visualized through the CCD camera. The equivalent and clear images of the word “∨” further demonstrate that the microholes have smooth surfaces and uniform profiles, including uniform microhole depths^26^,^27^. Furthermore, Fig. 3(b) illustrates that different diameters of microholes on both glass and Si substrates can be achieved by modulating the wet-etching time.

### Scanning electron microscopic images of the fabricated samples

**Figure 4:**
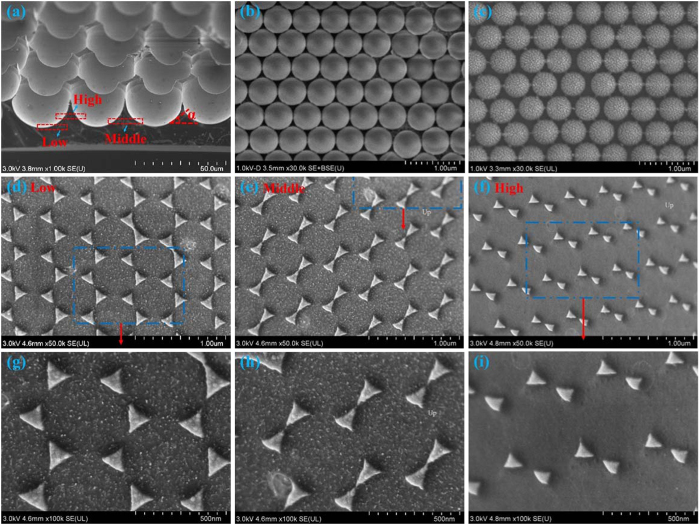
(**a**) ~60°-tilted SEM image of microholes etched ~18 min. Experimental top-view SEM images of PS spheres (**b**) before and (**c**) after plasma etching. Experimental top-view SEM images of quasi-3D bowtie nanoantennas on the (**d**) low, (**e**) middle and (**f**) high regions. Magnified SEM images of quasi-3D bowtie nanoantennas on the (**g**) low, (**h**) middle and (**i**) high regions. The blue dashed lines and word “up” represent the magnified and higher regions, respectively.

Figure 4 is a collection of the scanning electron microscopy (SEM) images of uniformly distributed microholes, polystyrene (PS) spheres and quasi-3D bowtie nanoantennas on Si substrates. As shown in Fig. 4(a), “α” represents the position angle between the tangent line of microholes and the horizontal line. In this paper, three regions are defined to simplify the investigation. The low, middle and high regions represent the regions with the “α” at the approximate ranges of [0, 20°], [20°, 40°] and [40°, 60°], respectively. Figure 4(b) and (c) show that uniform PS spheres can be achieved before and after oxygen plasma etching, which are scanned on a flat area. On the low region, Fig. 4(d) and (g) show the quasi-3D bowtie nanoantennas with the smallest gap size of ~70 nm, and the lengths of three sides are ~140 nm, ~135 nm and ~135 nm, respectively. The quasi-3D bowtie nanoantennas with the smallest gap size of ~15 nm on the middle region are illustrated in Fig. 4(e) and (h). The lengths of three sides are ~115 nm, ~150 nm and ~150 nm, respectively. For quasi-3D bowtie nanoantennas on the high region, the smallest gap size is ~55 nm, and lengths of three sides are ~75 nm, ~95 nm and ~95 nm, respectively. Therefore, when the angle α changes in different regions, the bowtie and gap sizes of quasi-3D bowtie nanoantennas are also changed.

### Electromagnetic results retrieved from simulations

**Figure 5:**
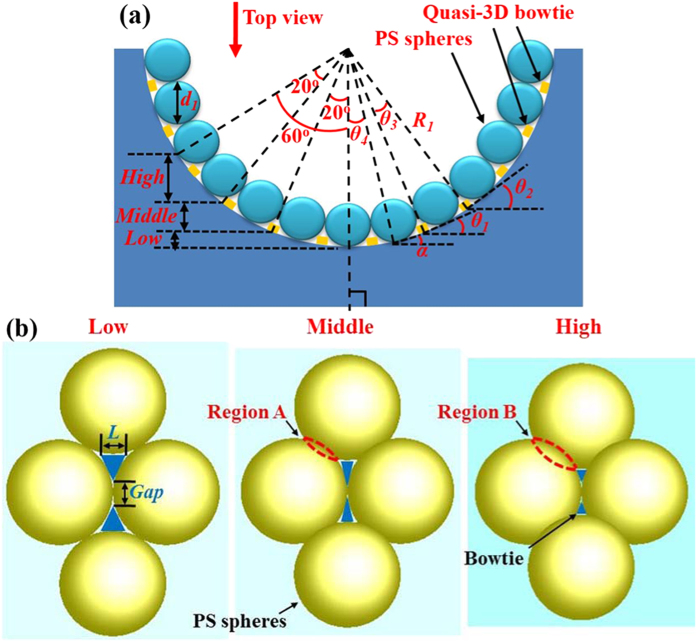
(**a**) Schematic of the cross section of a microhole. (**b**) Schematic illustrations of top-view images in different regions.

As illustrated in Fig. 5(a), “*R*_*1*_” and “*d*_*1*_” are the radius of microholes and the diameter of PS spheres, respectively. The bending angle “*θ*” [Fig. 1(a)] can be calculated as


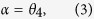



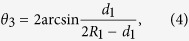



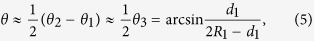


where the symbols *α* in Eq. (3), Figs 4(a) and 5(a) have the same meaning. The symbols “*θ*_*1*_” and “*θ*_*2*_” represent the angles between the tangent lines of adjacent quasi-3D bowtie nanoantennas and the horizontal lines. In Fig. 4, *R*_*1*_ and *d*_*1*_ are approximately 15 μm and 0.5 μm, respectively. According to Eqs (4) and (5), the bending angle *θ* can be found to be 0.97°. In this paper, as *θ* is small, it is set to be 0 to simplify the simulations. During the fabrication procedure, *R*_*1*_ can be tuned by etching time and *d*_*1*_ can be changed by different PS spheres. Therefore, the *θ* can be easily modulated by the fabrication processes. In addition, the low, middle and high regions defined in Fig. 4(a) are also schematically illustrated in Fig. 5. It has been clearly shown that, the length and gap sizes [Figs 4(d–f) and 5(b)] of quasi-3D bowtie nanoantennas in different regions are different from each other. From the top-view orientation to observe the microholes [Fig. 5(a)], the higher region PS spheres located, the larger overlapped area can be achieved [Region B is larger than Region A in Fig. 5(b)]. As the metal film was deposited along the top-view orientation, the overlapped areas are considered to be the main reason contributing to the different sizes of quasi-3D bowtie nanoantennas in different regions shown in Fig. 4(d–f).

In the simulation models shown in Fig. 1(b), the symbol “*β*” is set as *β* = 90°–*α*. The size of bowtie nanoantennas used in simulations is based on the SEM images shown in Fig. 4. To simplify the simulations, the angle “*β*” is set as 80°, 60° and 40° (the intermediate value of each region) for the metamaterial slabs at the low, middle and high regions, respectively.

**Figure 6:**
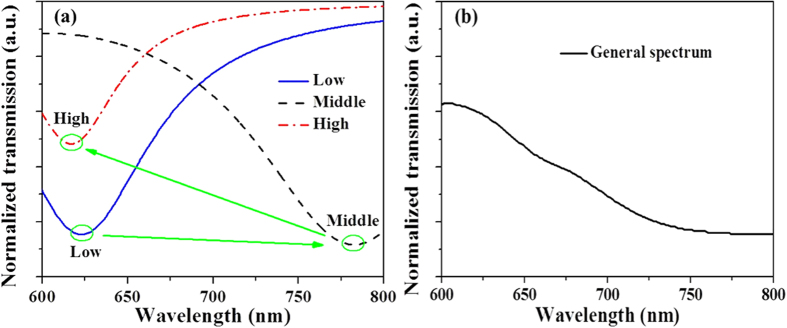
(**a**) Simulated transmission spectra of metamaterial slabs in different regions. (**b**) Experimental transmission spectrum for the average effect of the metamaterial slab.

As shown in Fig. 6(a), pronounced resonances of transmission spectra are presented at ~623 nm, ~783 nm and ~617 nm for metamaterial slabs at the low, middle and high regions in microholes, respectively, which are obtained by Eq. (2). The shifts of resonant ranges are mainly related to the length and gap sizes of bowtie nanoantennas^5^,^32^,^33^, as well as the position angle “*α*”. As the size of quasi-3D bowtie nanoantennas in the microholes (Fig. 4) can be gradually changed from the low to high regions, the resonance could be regarded as consecutive in the broad range from ~617 nm to ~783 nm, which is illustrated as the green arrows in Fig. 6(a). Figure 6(b) shows the measured transmission spectrum for the average effect of the metamaterial slab. It indicates that the metamaterial slab made of quasi-3D bowtie nanoantennas has the wide resonance in the range from around 600 nm to 800 nm, which may result from the consecutive resonances of quasi-3D bowtie nanoantennas in different regions of the microholes. As a result, both simulated and experimental results illustrate that the metamaterial slab made of quasi-3D bowtie nanoantennas has the resonance at the visible range. On the other hand, the diameters of the low, middle and high regions (less than 30 μm) in the microhole [Fig. 4(a)] are much smaller than that of laser facula (normally ~500 μm) during the transmission measurements by UV visible spectrophotometer. Therefore, it is very difficult to measure the transmission spectra accurately for different regions in a microhole, which needs to be further studied.

**Figure 7:**
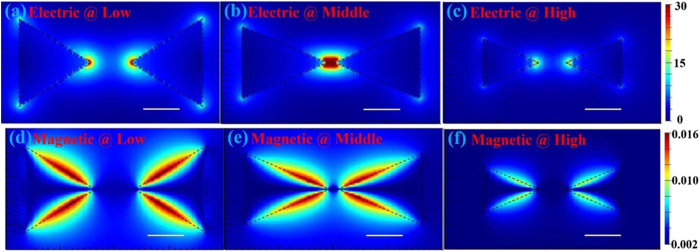
Simulated electric field distributions on metamaterial slab at (**a**) low, (**b**) middle, and (**c**) high regions. Simulated magnetic field distributions of metamaterial slab at (**d**) low, (**e**) middle, and (**f**) high regions. The white scale bar and black dashed lines represent 60 nm and contour profiles of bowtie nanoantennas, respectively.

Simulated electric and magnetic field distributions on metamaterial slabs made of bowtie nanoantennas at different regions are shown in Fig. 7. Different regions are monitored at the corresponding resonant wavelengths shown in Fig. 6(a). For the metamaterial slab, Fig. 7(a) and (d) are monitored at 623 nm in the low region, Fig. 7(b) and (e) are monitored at 783 nm in the middle region and Fig. 7(c) and (f) are monitored at 617 nm in the high region. As the intensities of electric fields in the tips of bowties are higher than those of the other parts, electric resonant couplings are mainly exited in the tips of bowtie nanoantennas. On the other hand, as illustrated in Fig. 7(d,e) and (f), magnetic intensities are mainly focused on the contours of bowtie nanoantennas, which indicates that magnetic resonant couplings could exit on the contours of bowtie nanoantennas. According to Maxwell’ equations, the rate of change of the magnetic flux is highest in the tips of bowtie nanoantennas, while rate of change of electric field on the contours of bowtie nanoantennas is much higher than those on other positions. Therefore, both electric and magnetic resonances could be obtained in these quasi-3D bowtie nanoantennas with a broad resonant range, which are promising to be applied in 3D metamaterial applications.

**Figure 8:**
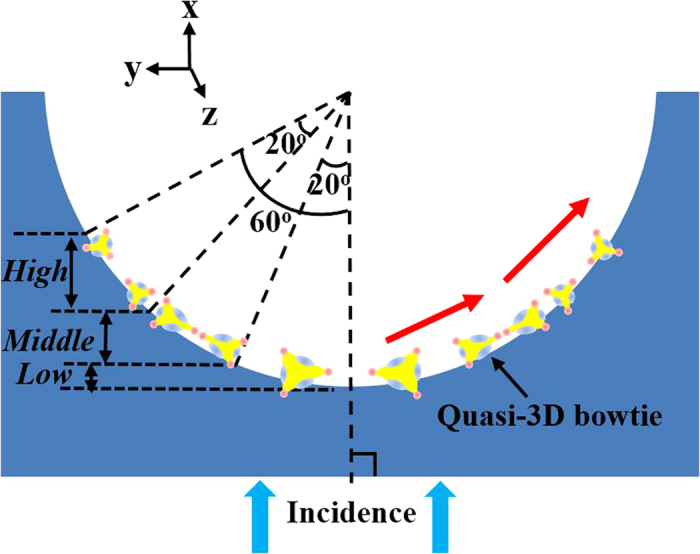
Schematic illustrations of electrical and magnetic distributions in different regions. In the bowtie nanoantennas, the light blue and red colors represent the magnetic and electrical couplings, respectively.

Figure 8 schematically shows the EM distributions of quasi-3D bowtie nanoantennas. When EM waves propagate from the bottom, electric resonant couplings are mainly focused in the tips of bowtie nanoantennas, while magnetic intensities are mainly focused on the contours of bowtie nanoantennas. As the red lines shown in Fig. 8, the EM intensities can be transferred from the bottom to the top of microholes, indicating that quasi-3D bowtie nanoantennas could modulate the resonance along the x, y and z directions.

## Discussion

We have fabricated a novel array of quasi-3D bowtie nanoantennas with a low-cost and facile method. The periodic size and diameter of microhole arrays can be easily tuned by the laser patterning combined with wet etching, which is beneficial to achieve certain nanostructures under different conditions. According to the simulated results, the electric and magnetic resonances are obtained in the tips and contours of quasi-3D bowtie nanoantennas, respectively, which is beneficial for metamaterial fabrications. As the size and gap of quasi-3D bowtie nanoantennas is gradually changed from the low to high regions in the vertical direction, the broad resonant range for the monolithic quasi-3D bowtie arrays could be achieved by different electric and magnetic resonancs at visible wavelengths. Finally, the ability to fabricate quasi-3D bowtie nanoantennas by itself is of great potential significance to some metamaterial applications.

## Methods

### Fabrication procedure

**Figure 9:**
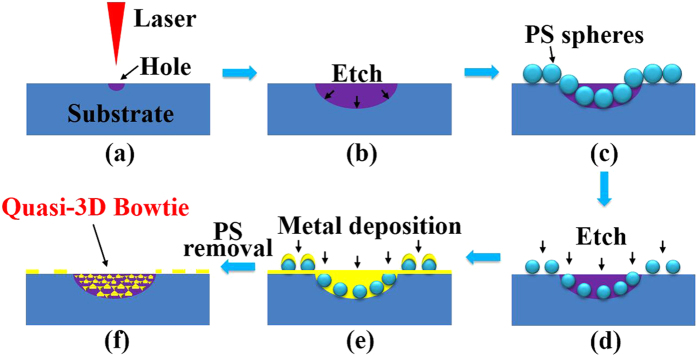
Schematic of the processes for fabricating quasi-3D bowtie nanoantennas. (**a**) Laser irradiation. (**b**) Wet etching process. (**c**) PS spheres self-assembled on the substrate. (**d**) PS etching process. (**e**) Deposit metal film on the substrate. (**f**) PS sphere removal.

Figure 9 is a schematic of the fabrication procedure for making samples with quasi-3D bowtie nanoantennas. Firstly, microhole arrays with diameters of less than 15 μm were produced by a nanosecond laser with a central wavelength of 355 nm [Fig. 9(a)]. The substrate was mounted on a translation stage controlled by a computer program and the focal position in the z direction was not changed in the experiments. During the irradiating process, the exposure holes were generated point-by-point. After laser patterning, the samples with microhole arrays were ultrasonically cleaned by acetone, alcohol, and deionized water for 10 min each. Next, the samples were etched in a mixed acid solution at room temperature [Fig. 9(b)]. In this step, we use the etching time to control the microhole size. When concave surfaces were successfully fabricated, the samples were cleaned by deionized water and dried. In the stage of Fig. 9(c), a hexagonally-ordered monolayer template of 500-nm-diameter polystyrene (PS) spheres was coated onto the cleaned samples by the self-assembly method. Then, the dried samples were etched by oxygen plasma for ~50 s to reduce the PS diameters [Fig. 9(d)]. After that, a 30-nm-thick gold (Au) film was deposited on the samples by electron beam evaporation [Fig. 9(e)]. Lastly, the chloroform solution was utilized to remove the PS spheres. Quasi-3D bowtie nanoantennas are able to be obtained in the microholes [Fig. 9(f)], as the surfaces of microholes are not flat. In the fabrication processes, the glass or silicon (Si) wafers were used as the substrates. For the glass substrate, the acid solution used in Fig. 9(b) was ~8% hydrofluoric (HF) acid solution diluted by deionized water. When the substrate was Si wafer, the acid solution consisted of ~5% HF acid solution and ~25% nitric acid (HNO_3_) solution diluted by deionized water. In the etching process, the HNO_3_ solution can oxidize the Si wafer and the HF solution can remove the Si oxides.

## Additional Information

**How to cite this article**: Zhao, Y. *et al*. Metamaterial study of quasi-three-dimensional bowtie nanoantennas at visible wavelengths. *Sci. Rep.* **7**, 41966; doi: 10.1038/srep41966 (2017).

**Publisher's note:** Springer Nature remains neutral with regard to jurisdictional claims in published maps and institutional affiliations.
